# A THREE-TIER EXAMINATION OF RACIAL DISPARITIES IN PAIN AT THE END OF LIFE

**DOI:** 10.1093/geroni/igad104.1844

**Published:** 2023-12-21

**Authors:** Zainab Suntai, Susanny Beltran, Elyse McMullen

**Affiliations:** Baylor University, Waco, Texas, United States; University of Central Florida, Orlando, Florida, United States; Baylor University, Waco, Texas, United States

## Abstract

Over the past few decades, research focused on improving end-of-life outcomes has burgeoned within the field of gerontology. Pain is one of the most common symptoms assessed at the end of life and research has shown that Black and Hispanic older adults are significantly more likely to experience pain compared to White older adults. However, few studies have sought to describe racial/ethnic differences in help-seeking behavior for pain and satisfaction with pain management among older adults. This study employed a three-tier approach by investigating 1) differences in the experience of pain at the end of life, 2) differences in help-seeking behavior for pain at the end of life, and 3) differences in satisfaction with pain management at the end of life. Data were derived from the combined 2012 to 2020 last-month-of-life interviews as part of the National Health and Aging Trends Study. Using a multivariate logistic regression model, results showed that Black and Hispanic older adults were more likely to experience pain at the end of life, less likely to have received help for their pain, and less likely to report that they were satisfied with the level of help received. These results point to a disparity in not only the level of pain experienced at the end of life, but the treatment and quality of pain management. Implications for aging research, policy, and practice are discussed.

